# Correction of Temperature Variation with Independent Water Samples to Predict Soluble Solids Content of Kiwifruit Juice Using NIR Spectroscopy

**DOI:** 10.3390/molecules27020504

**Published:** 2022-01-14

**Authors:** Harpreet Kaur, Rainer Künnemeyer, Andrew McGlone

**Affiliations:** 1 The Dodd Walls Centre for Photonic and Quantum Technologies, School of Engineering, The University of Waikato, Hamilton 3216, New Zealand; 2 The New Zealand Institute for Plant and Food Research Limited, Ruakura, Hamilton 3214, New Zealand; Andrew.McGlone@plantandfood.co.nz; 3 The Dodd Walls Centre for Photonic and Quantum Technologies, The University of Otago, Dunedin 9054, New Zealand; r.kunnemeyer@gmail.com

**Keywords:** soluble solids content, Brix, kiwifruit juice, aquaphotomics, near infrared spectroscopy, extended multiplicative scatter correction (EMSC), external parameter orthogonalisation (EPO)

## Abstract

Using the framework of aquaphotomics, we have sought to understand the changes within the water structure of kiwifruit juice occurring with changes in temperature. The study focuses on the first (1300–1600 nm) and second (870–1100 nm) overtone regions of the OH stretch of water and examines temperature differences between 20, 25, and 30 °C. Spectral data were collected using a Fourier transform–near-infrared spectrometer with 1 mm and 10 mm transmission cells for measurements in the first and second overtone region, respectively. Water wavelengths affected by temperature variation were identified. Aquagrams (water spectral patterns) highlight slightly different responses in the first and second overtone regions. The influence of increasing temperature on the peak absorbance of the juice was largely a lateral wavelength shift in the first overtone region and a vertical amplitude shift in the second overtone region of water. With the same data set, we investigated the use of external parameter orthogonalisation (EPO) and extended multiple scatter correction (EMSC) pre-processing to assist in building temperature-independent partial least square regression models for predicting soluble solids concentration (SSC) of kiwifruit juice. The interference component selected for correction was the first principal component loading measured using pure water samples taken at the same three temperatures (20, 25, and 30 °C). The results show that the EMSC method reduced SSC prediction bias from 0.77 to 0.1 °Brix in the first overtone region of water. Using the EPO method significantly reduced the prediction bias from 0.51 to 0.04 °Brix, when applying a model made at one temperature (30 °C) to measurements made at another temperature (20 °C) in the second overtone region of water.

## 1. Introduction

Water is the major constituent of fruits, typically more than 80% [1,2], and absorbs near-infrared (NIR) radiation [3,4]. The NIR spectrum of fruit shows a strong absorption peak around 970 nm, which corresponds to the second overtone of the OH stretch in water [5,6]. NIR spectroscopy (NIRS) models for predicting dry matter (DM) and soluble solids content (SSC) of fruit (apples) have been developed using the narrow spectral range from 800 to 1100 nm around this absorption peak [5].

NIRS models are called robust when their prediction accuracy is relatively insensitive to unknown changes in external factors [7]. A factor that can strongly affect NIR model performance is temperature [8]. Shifts in the water absorbance bands with temperature can reduce model performance [9,10,11,12]. Acharya et al. [12] have studied the effect of temperature on prediction models of fruit quality, observing that a calibration equation developed at one fixed temperature could not reliably predict on samples measured at a different temperature. Roger et al. [10] found a model offset bias of 8 °Brix for a temperature variation of 20 °C (range 5–25 °C) for SSC prediction in apples. Several techniques have been previously investigated to compensate fruit model predictions for fluctuations in sample temperature. Kawano et al. [9] developed calibration equations for peaches using samples at different temperatures. Peirs et al. [13] similarly developed robust calibration models for a wide range of apple cultivars, incorporating samples at all temperature ranges expected in future measurements. Roger et al. [10] removed the temperature-induced bias in SSC predictions on apples by applying the external parameter orthogonalisation (EPO) algorithm, as a pre-processing step, to remove the part of the spectral data matrix most affected by temperature. Several new techniques have been reported recently in the literature, indicating the problem is far from solved for all circumstances [14].

The framework of aquaphotomics appears suitable for examining the temperature sensitivity of fruit spectra. Aquaphotomics is an NIR spectral analysis methodology that focuses on changes in the pattern of water absorbance bands due to perturbations by extraneous factors. The field has aided the understanding of the role of water in biological systems [15,16,17,18]. The effect of perturbations on water binding structures has been observed due to variation in solute concentration, temperature, and other environmental factors [16,18,19]. The aquaphotomics methodology defines 12 water absorption bands in the first overtone region of water, called the water matrix coordinates (WAMACS), which describe the water states in an aqueous system. The methodology has been applied to the study of apple juice when the sample temperature is increased from 20 to 30 °C, revealing an increase in free water molecule states that raises the spectral absorbance at 1414 nm [20]. 

In this paper, we use the aquaphotomics approach to study the changes in the water structure of kiwifruit juice caused by variation of temperature in the vicinity of the 1450 nm (first overtone of OH stretch of water) and 970 nm (second overtone of OH stretch of water) absorbances. The second overtone region has had very little research attention from an aquaphotomics perspective, which is surprising given the importance of that region for intact fruit quality prediction by NIR, including on kiwifruit [21,22]. Physically filtered fruit juices, removing most particulate matter, minimize light scattering variation between samples and thus provide an ideal medium for fundamental aquaphotomics studies involving controlled temperature perturbations of the sample water chemistry. The results from such studies, if clarifying the fundamental mechanisms involved, may help in understanding and/or overcoming the temperature sensitivities associated with NIRS whole fruit measurements. 

We also evaluate the pre-processing modeling methods, extended multiplicative scatter correction (EMSC), and EPO, using pure water spectra as interferent, to minimize the temperature sensitivity of NIRS models for the SSC of kiwifruit juice. Previous aquaphotomics analysis showed the effective use of EMSC for minimizing the temperature sensitivity of apple juice models for SSC prediction, where the spectral measurements were over the 1300–1600 nm first overtone region, and the required interferent spectra were derived from pure water measurements [20]. The EPO technique is somewhat similar in requiring the specification of an interferent spectrum. It has been previously explored for the development of temperature-insensitive NIR models of intact apple SSC across the second overtone region using common fruit samples, measured at different temperatures to derive the required interferent spectra [10].

## 2. Materials and Methods

### 2.1. Sample Preparation

A total of 100 fully ripe Zespri^®^ SunGold Kiwifruit (*Actinidia chinensis* var. *chinensis* ‘Zesy002’) were purchased from New Zealand retail stores. Juice was expressed from about 2 cm thick endcaps, removed from the stem and calyx ends of each fruit, and was collected in Eppendorf tubes. The samples were centrifuged at 13,400 rpm for 3 min (MiniSpin, Eppendorf, Hamburg, Germany) and then filtered through a 0.2 μm syringe filter to produce a clear juice (Figure 1). The samples were stored in a refrigerator at 4 °C. Fourier transform–near-infrared (FT-NIR) spectra and reference SSC measurements were performed the next day after the samples were equilibrated to room temperature (20 °C). Milli-Q water with a resistivity of 18.2 MΩ cm was produced using a water purification system (Millipore, Thermofisher Scientific, Knox, Australia). 

### 2.2. Reference SSC (°Brix) Measurement

The SSC value of the kiwifruit juice samples was measured at room temperature using a digital refractometer (PAL-1, Atago Co., Ltd., Tokyo, Japan), calibrated with Milli-Q water. The Brix value was recorded after placing approximately 0.5 mL of juice into the measurement chamber of the refractometer; this was enough to fully cover the optical interface. 

### 2.3. FT-NIR Spectral Measurements

Transmittance spectra of the juice samples were measured at 20, 25, and 30 °C (±1 °C) with an FT-NIR spectrometer (Tango, Bruker Corporation, Bremen, Germany) equipped with a temperature-controlled holder. Two measurements were acquired for each juice sample, using quartz cuvettes of 1 mm and 10 mm optical path length for the 1300–1600 nm and 870–1100 nm wavelength ranges, respectively [23]. For each measurement, one spectrum was the average of 32 successive scans and was recorded with a resolution of 16 cm^−1^. The total number of juice spectra was 600 (100 samples × 1 consecutive scan × 3 temperatures × 2 cuvettes). The samples were divided into two sets, one for the 1300–1600 nm and one for the 870–1100 nm wavelength ranges. After the removal of five outliers, anomalous readings speculated to be laboratory blunders, the final data set consisted of 95 juice samples (285 spectra for three temperatures) in each wavelength set. The spectral region above 1800 nm was discarded because of the high absorption in aqueous samples. To monitor interfering signals, a reference spectrum of Milli-Q water was taken at the beginning, middle, and end of the experiment, which resulted in a total of 18 water spectra (3 samples × 1 consecutive scan × 3 temperatures × 2 cuvettes).

### 2.4. Aquaphotomics Analysis

Aquaphotomics water matrix coordinates (WAMACS) were created using the peak wavelengths identified in a principal component analysis (PCA) of the full data set of fruit juice spectra over the three temperatures in the first and second overtone regions of the OH stretch of water. An anharmonic oscillator model was used to establish 12 water bands in the second overtone region that corresponded to the previously established wavelengths in the first overtone region of water [24]. Aquagrams displaying the resulting water spectral pattern (WASP) in each overtone region were studied to observe the effect of temperature variation.

### 2.5. Multivariate Analysis

Predictive models were developed using MATLAB version R2018b (MathWorks Inc., Natick, MA, USA) and the PLS toolbox version 8.6.2 (Eigenvector Research Inc., Wenatchee, WA, USA). The analysis involved the development of predictive models using spectra pre-processed by:

#### 2.5.1. SNV + 2D

This is the standard normal variate transformation of the raw spectra followed by second derivative processing (Savitzky–Golay second-order derivative with smoothing parameters: width 15, order 2).

#### 2.5.2. EMSC

This is the extended multiplicative scatter correction of the raw spectra. The concept of EMSC pre-treatment was introduced by Martens et al. [25,26]. EMSC was designed to remove chemical variabilities by using a model framework that segregates the spectral response of the analyte of interest from that of a known interference. 

Equation (1) describes the theory of EMSC:

(1) $$X=b_{0\;}+b_{1}\bar{X}+b_{2}I+e$$

where *X* is the raw observed spectra, $\bar{X}\;$ is the mean spectrum (the mean of all calibration spectra), *I* is an interferent spectrum (to be determined), *b*_0_, *b*_1_, and *b*_2_ are fitting constants, and *e* is the residual [18]. Rearranging Equation (1) leads to:

(2) $$\frac{X-b_{0}}{b_{1}}-\frac{b_{2}I}{b_{1}}=\bar{X}+\frac{e}{b1}$$

The left-hand side of Equation (2) defines the corrected spectra,

(3) $$\hat{X}=\frac{X-b_{0}}{b_{1}}-\frac{b_{2}I}{b_{1}}$$

where the constant terms can be estimated by multiple linear regression (MLR).

#### 2.5.3. EPO

This is the external parameter orthogonalisation of the raw spectral matrix **X**. The concept of EPO was introduced by Roger et al. [10]. It is a pre-processing method that aims at removing the part of the **X** matrix space most influenced by the external parameter variations. The method identifies the parasitic subspace for removal by computing a PCA on a small set of spectra measured on the same objects, while the external parameter is varying. 

The theory of the EPO algorithm is outlined below [27]. 

The spectra matrix **X** (size n × m) can be written as:

**X** = **XP** + **XQ** + **R** (4)

where **P** is the projection matrix (size m × m) of the useful part of the spectra: X* = **XP**; **Q** is the projection matrix (size m × m) of the not useful part (e.g., influenced by temperature) of the spectra: **X#** = **XQ**; **R** is the residual matrix (size n × m); n is the number of samples, and m is the number of wavelengths.

The aim of EPO is to obtain the useful spectra X* = **X** (**I** − **Q**), while matrix **Q** can be written as **Q** = **GG^T^** where **G^T^** is the transpose of **G**. The transformed spectra for both the calibration and validation sets are then calculated as X* = **XP** where **P** = **I** − **GG^T^**, and **I** is the identity matrix. To estimate **G**, the uninformative part of the spectra that is orthogonal to the useful part of the spectra, the principal component of the difference spectra **D** is calculated. **D** is the difference matrix generated by subtracting the average spectra for the samples at the lowest temperature (in our case) from the samples at all temperatures.

#### 2.5.4. All Temperature Method

This involved combining samples from all three temperatures and applying a standard pre-treatment on all spectra, both calibration and validation, of SNV transformation followed by 2nd derivative processing.

### 2.6. Statistical Analysis

The main data set in the long-wavelength region (1300–1600 nm) and the short wavelength region (870–1100 nm) was split into three subsets for 20, 25, and 30 °C temperature, respectively. The samples were first rank ordered by SSC value and then systematically split into ten different groups, using a Venetian blind selection approach, delivering SSC equivalence between the groups. This arrangement enabled a 10-way leave-each-group-out approach to calibration-validation set modeling and analysis. Each of the ten calibration–validation sets were created by holding out a single group in turn, as an independent validation data set, and leaving the remaining nine groups to be combined as the calibration data set. Consequently, the total number of samples in each calibration set was 71, and in each validation set was 24. A separate 10-way Venetian blind cross-validation process was also undertaken with the calibration modeling on each calibration data set. 

## 3. Results and Discussion

The SSC of kiwifruit juice ranged from 11.9 to 19.2 °Brix, with a mean of 16.54 °Brix and a standard deviation of 1.26 °Brix. Figure 2 shows the distribution of SSC for all fruit juice samples in the experiment. There was a very small number of relatively low SSC samples, below 14 °Brix, which may be population outliers.

### 3.1. The Raw Spectra

The absorbance plot in Figure 3 illustrates that as the temperature increased in kiwifruit juice, the absorbance curve in the first overtone region shifted to shorter wavelengths with a broadening of the peak and a decrease in intensity (Figure 3a). However, in the second overtone region, there was a slight upward shift in intensity towards the shorter wavelengths with increasing temperature (Figure 3b). The isosbestic points were 1444 nm (before the water peak wavelength) and 994 nm (after the water peak wavelength), each approximately 4 nm away from reported pure water isosbestic points at 1440 nm and 990 nm, respectively [28].

### 3.2. Aquaphotomics Analysis

The wavelengths of the peak and trough in the PC1 spectrum (from PCA on all juice samples and temperatures) were at 1414 nm and 1494 nm (the first overtone), and 963 nm and 1027 nm (the second overtone), as shown in Figure 4a,b. These wavelength pairs correspond to C5 (S_0_: free water) and C11 (S_4_: species with four hydrogen bonds) activation for the first overtone, and C6 (water hydration) and C12 (strongly bonded water) activation for the second overtone region. The remaining WAMACS assignments, not directly identified from the PCA, were selected as the midpoints of each known water band in Table 1. As expected, the zero-crossing points for the two PC1 plots were identical to the isosbestic points observed in Figure 3.

### 3.3. Aquagrams

The aquagrams of average spectra of the juice at three temperatures are illustrated in Figure 5a,b for the two overtone regions. There are strong similarities in the two overtone regions, free water species increasing with temperature as the water structure becomes less organized as a result of increased molecular motion and less stable H-bonds. However, there is a difference with the asymmetric stretching and bending (ν_2_ + ν_3_) only observed to increase with temperature for the second overtone region. We might have expected that the same water coordinates to be similarly highlighted in the first and second overtone region. However, this is not quite the case here.

### 3.4. EMSC Correction

#### Pure Water Analysis

When applying PCA to the water spectra, the shape of the PC1 loading in the first overtone region (Figure 6) is very similar to that reported by Segtnan et al. [8] and Maeda et al. [31], indicating a change in water structure due to a change in temperature. The shape of the PC1 loading of water in the second overtone region (Figure 6) is similar to the PC1 loading in the first overtone region. Hence, the respective PC1 loadings, for the first overtone and second overtone regions were used as the interferent spectra in the EMSC correction method (Equation (3)).

### 3.5. EPO Correction

#### PCA of the Difference Matrix D for EPO Correction

There was a variation in the temperature around the peak wavelength region of the juice spectra (Figure 3). When applying PCA to the difference matrix, D, of water and juice spectra (Figure 7), the shape of the PC1 loadings (Figure 8a,b) were nearly identical, and the peak and trough positions were identical to those determined in the PCA of the raw juice and water spectra (Figure 4 and Figure 6). Therefore, the PC1 loadings of the difference matrix, D, of water were used in the EPO correction (Figure 6) as the interferent spectra to correct juice spectra against temperature variation.

### 3.6. Prediction of SSC

Application of both the EMSC and EPO pre-processing techniques generally reduced the SSC prediction bias by a large margin in both the first (Figure 9) and second (Figure 10) overtone regions, especially compared with SNV + 2D pre-processing. Beyond that, and particularly comparing EMSC and EPO, it is difficult to see any consistent trends or patterns in the results. For instance, in the first overtone region, the EMSC method seems advantageous (lower bias) compared with the EPO method when applying a model calibrated at 30 °C to a validation set at 20 or 25 °C (Figure 9c). However, that does not apply in reverse, a model calibrated at 20 °C is perhaps slightly better under the EPO method when applied to validation sets at 25 and 30 °C. In the second overtone region, the EPO method seems to have the advantage, although oddly it fails badly, even compared with the SNV + 2D method, when using a model calibrated at 25 °C on a validation set at 30 °C (Figure 10b). The variation in results of any particular method is relatively large, represented by standard deviation error bars in the graphs, and suggests a large amount of modeling noise between the various combinations of calibration and validation data sets.

The performance of each method is shown in detail in Table 2 and Table 3. The result for the All-Temperature method is listed for comparison. The all-temperature method resulted in the lowest overall biases of 0.03 in the first overtone region and less than 0.06 in the second overtone region of water. However, the EPO method consistently produced the lowest SEP and RMSECV for the first and second overtone regions of water. The RMSECV errors in the first and second overtone regions were fairly consistent, approximately 0.09 and 0.12 respectively when using the EPO method for calibration models at 20, 25, and 30 °C. However, the bias results were not consistent. For example, the calibration model at 30 °C predicting at 20 °C, produced a bias of 0.23 in the first overtone region whereas a bias of 0.04 was generated in the second overtone region. The best choice of wavelength region may depend on other matters, such as sample thickness—analysis of thicker samples, as in thicker than the 1 mm pathlength, may demand the use of the second overtone region of water to achieve sufficient light transmission.

## 4. Conclusions

The aquaphotomics study here has revealed that the free water components of kiwifruit juice increase and the bound water components decrease as the temperature rises from 20 to 30 °C. The key aquaphotomics wavebands in the first and second overtone regions were identified. The influence of increasing temperature on the peak absorbance of kiwifruit juice spectra was a lateral (wavelength) shift in the first overtone region and a vertical shift in the second overtone region of water. In the second overtone region, the C8 asymmetric stretching and bending component (ν_2_ + ν_3_) became more prominent in the aquagram with increasing temperature, which was not the case in the first overtone region.

Predictive modeling of the SSC of kiwifruit juice over the temperature range 20 to 30 °C was more robust (lower offset bias) when using EPO and EMSC pre-processing with an interference term generated from PCA of a simple and independent pure water-temperature spectral matrix experimentally generated over the same temperature range. The water-temperature matrix only needs to be created once and, in being independent of the kiwifruit juice samples, considerably simplifies the generation of temperature-independent predictions. The consequence of this is that model calibration data on actual juice samples need only be measured at one temperature, the EPO and/or EMSC pre-processing enables application at any other temperature within the measured temperature range. This approach may apply to other applications, such as other fruit juices or intact fruit modeling problems, where robustness against temperature changes is desirable. 

## Figures and Tables

**Figure 1:**
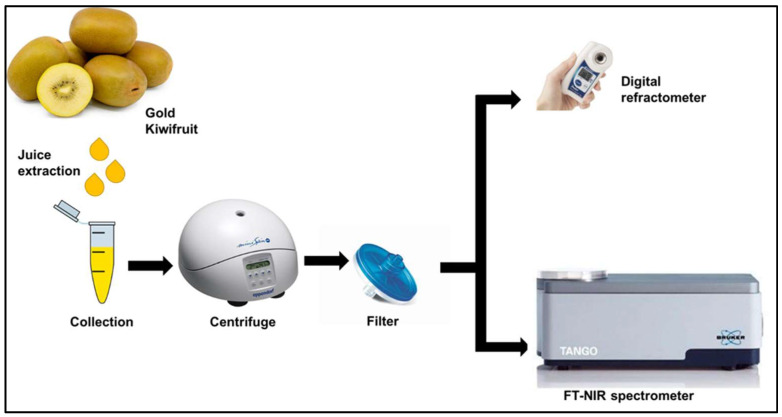
Experimental procedure for kiwifruit soluble solids content (SSC) measurement.

**Figure 2:**
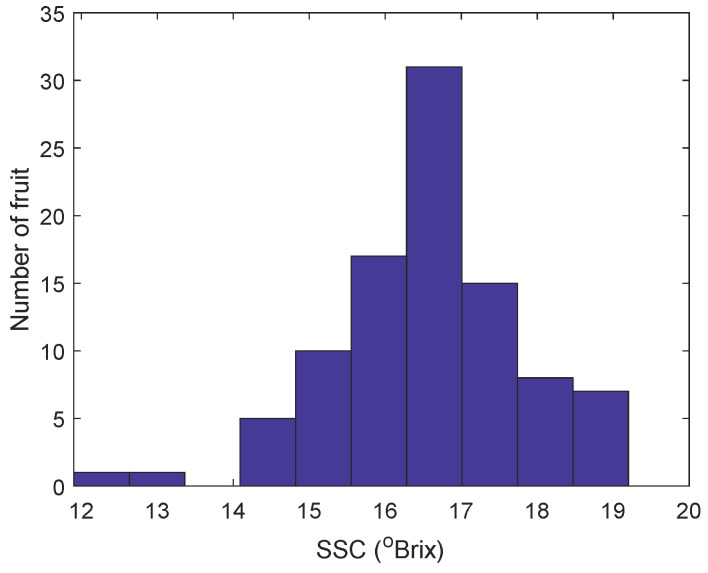
Number of fruit vs SSC for the kiwifruit juice samples.

**Figure 3:**
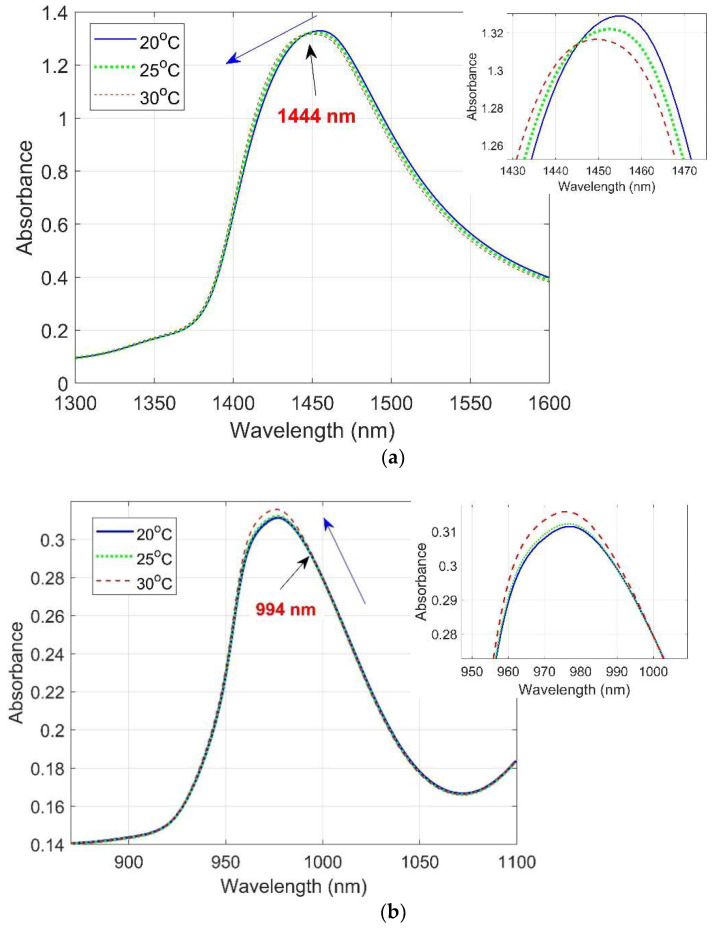
Average raw absorbance spectra of kiwifruit juice at three temperatures 20, 25, and 30 °C in (**a**) the first overtone and (**b**) the second overtone region of the OH stretch of water. Labels indicate wavelengths of the isosbestic points.

**Figure 4:**
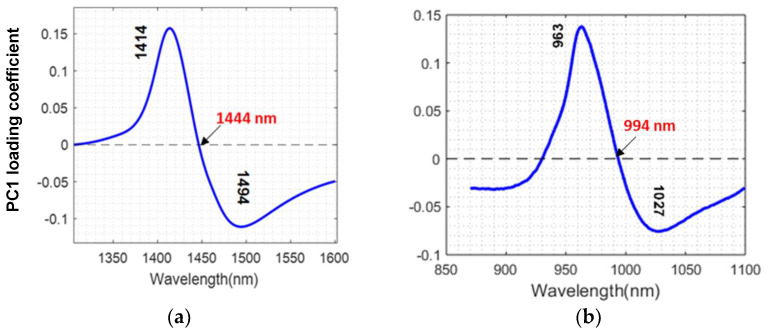
PC1 loading of kiwifruit juice in (**a**) the first overtone and (**b**) the second overtone region of the OH stretch of water. Labels indicate peak wavelengths (black) and zero-crossing points (red).

**Figure 5:**
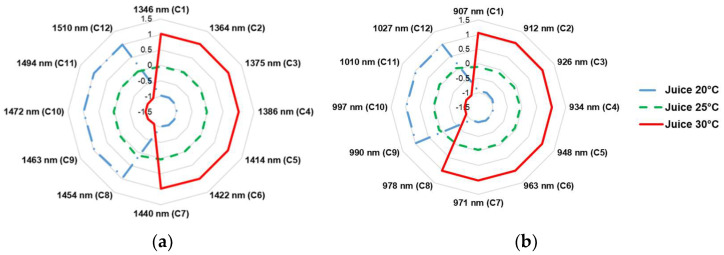
Aquagrams at three temperatures in (**a**) the first overtone (1300–1600 nm) (**b**) the second overtone (870–1100 nm) region of the OH stretch of water in kiwifruit juice.

**Figure 6:**
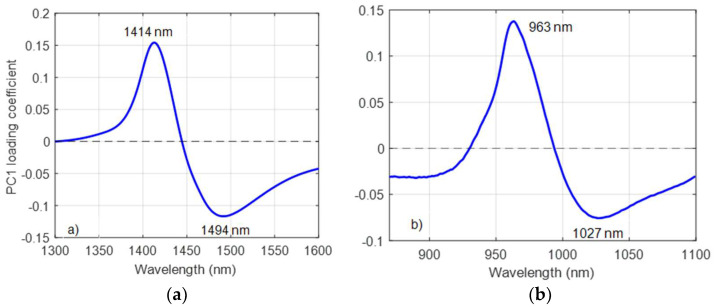
PC1 loading of water in (**a**) the first overtone and (**b**) the second overtone region of the OH stretch of water. Labels indicate peak and trough wavelengths.

**Figure 7:**
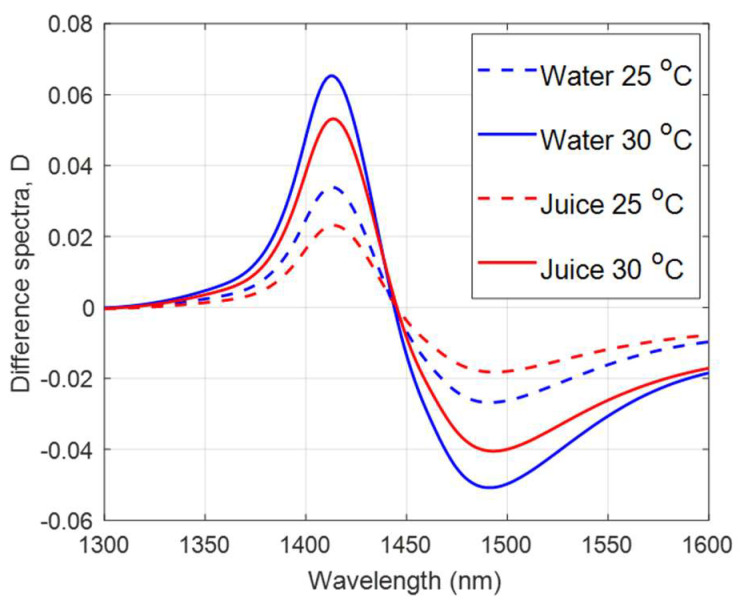
Average raw absorbance difference spectra of water and kiwifruit juice after subtracting average raw absorbance spectrum of water and juice at 20 °C, respectively.

**Figure 8:**
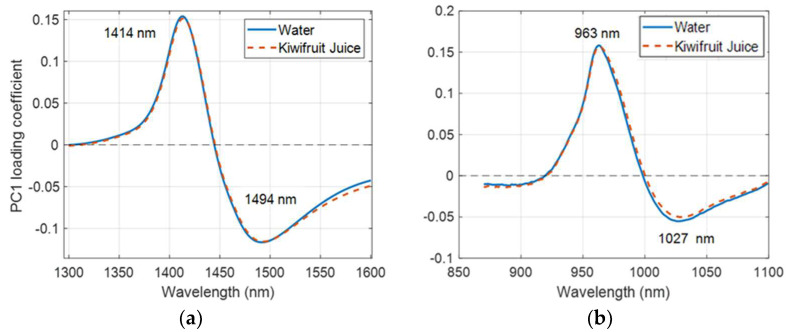
PC1 loading of water and juice difference matrix in the (**a**) 1300–1600 nm region with a 1 mm cuvette; and (**b**) 870–1100 nm region with a 10 mm cuvette. Labels indicate the peak and trough wavelengths.

**Figure 9:**
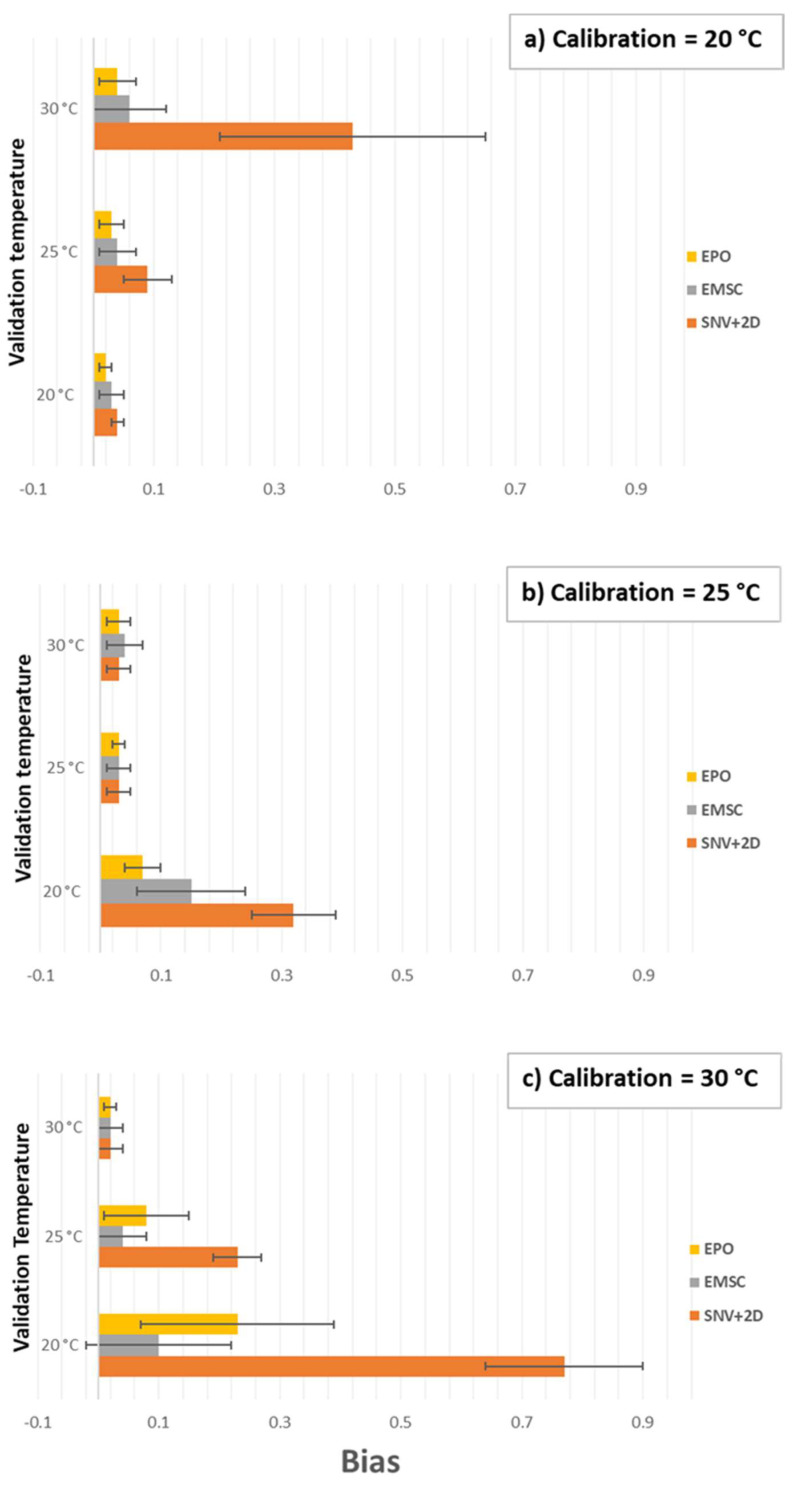
Validation temperature vs. bias at for SSC prediction of kiwifruit juice in the first overtone (1300–1600 nm) region of the OH stretch of water at calibration temperature (**a**) 20 °C, (**b**) 25 °C, and (**c**) 30 °C. Error bars show standard deviation.

**Figure 10:**
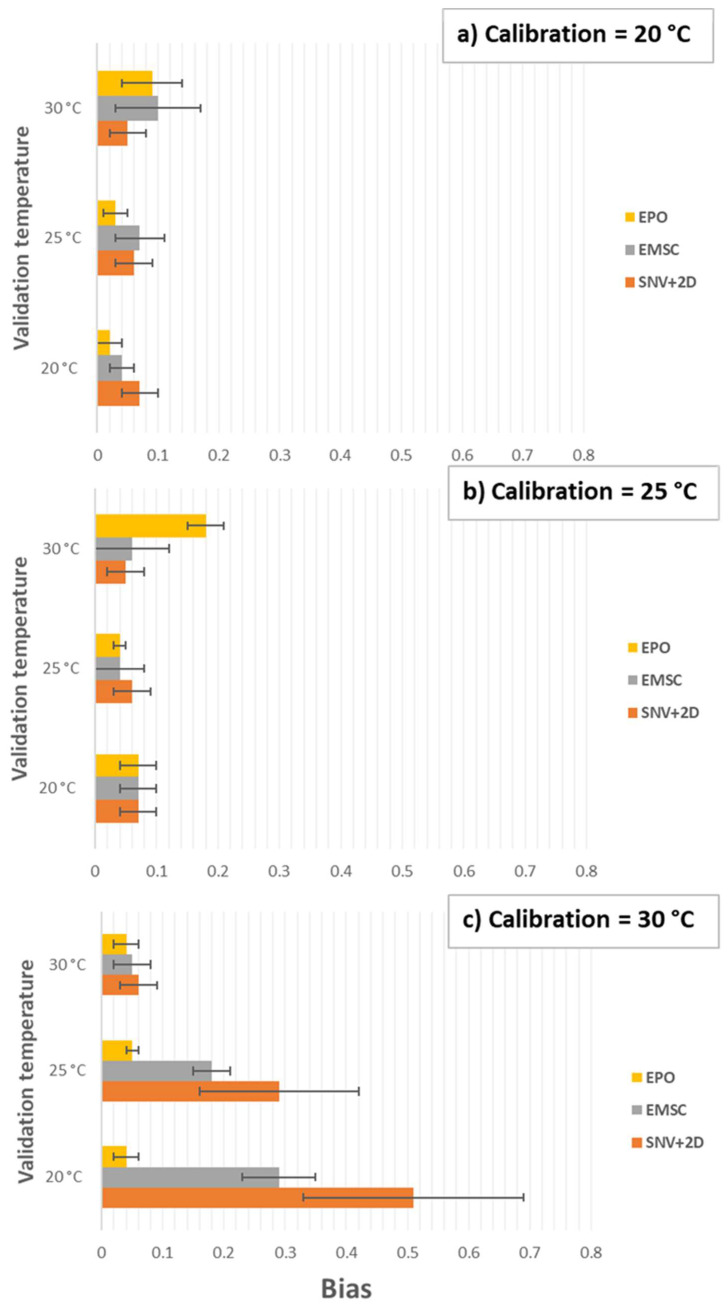
Validation temperature vs. bias for SSC prediction of kiwifruit juice in the second overtone (870–1100 nm) region of the OH stretch of water at calibration temperature (**a**) 20 °C, (**b**) 25 °C, and (**c**) 30 °C. Error bars show standard deviation.

**Table 1:** Temperature-perturbed water wavelengths of kiwifruit juice in the first [16,29] and the second overtone regions of OH stretch of water [30].

WAMACS	Assignment	Wavelengths in Overtone Region		Activated Wavelengths, nm	
		First (1300–1600 nm)	Second (800–1100 nm)	First Overtone	Second Overtone
C1	ν_3_—asymmetric stretching vibration	1336–1348	900–908		
C2	OH stretch—water solvation shell)	1360–1366	916–920		
C3	ν_1_ + ν_3_—H_2_O symmetric stretching and asymmetric stretching vibration	1370–1376	923–927		
C4	OH stretch (water solvation shell)	1380–1388	930–935		
C5	S_0_ (free water)	1398–1418	942–955	1414	
C6	Water hydration, H_5_O_2_	1421–1430	957–963		963
C7	S_1_—water molecules with 1 hydrogen bond	1432–1444	965–973		
C8	ν_2_ + ν_3_—H_2_O bending and asymmetric stretching vibration	1448–1454	975–979		
C9	S_2_—water molecules with 2 hydrogen bonds	1458–1468	982–989		
C10	S_3_—water molecules with 3 hydrogen bonds	1472–1482	992–998		
C11	S_4_—water molecules with 4 hydrogen bonds	1482–1495	998–1007	1494	
C12	Strongly bonded water or ν_1_, ν_2_	1506–1516	1014–1021		1027

**Table 2:** Performance comparison for soluble solids content (SSC) prediction of kiwifruit juice in the first overtone (1300–1600 nm) region of the OH stretch of water at different temperatures.

With 1 mm Cuvette in the First Overtone Region (1300–1600 nm)								
N_cal_ = 72, N_val_ = 23								
Calibration				Validation				
T_cal_ [°C]	Method	r^2^_cv_	RMSECV	T_val_ [°C]	r^2^_p_	RMSEP	BIAS	SEP
All	SNV + 2D	0.99	0.12 (±0.00)	20	0.99	0.12 (±0.01)	0.03 (±0.02)	0.12 (±0.01)
				25	0.99	0.12 (±0.02)	0.03 (±0.01)	0.12 (±0.02)
				30	0.99	0.13 (±0.02)	0.03 (±0.01)	0.12 (±0.02)
20	SNV + 2D	0.99	0.14 (±0.01)	20	0.98	0.16 (±0.01)	0.04 (±0.01)	0.15 (±0.01)
				25	0.96	0.24 (±0.05)	0.09 (±0.04)	0.22 (±0.05)
				30	0.98	0.48 (±0.21)	0.43 (±0.22)	0.19 (±0.02)
	EMSC	0.99	0.14 (±0.01)	20	0.99	0.13 (±0.02)	0.03 (±0.02)	0.13 (±0.01)
				25	0.98	0.15 (±0.02)	0.04 (±0.03)	0.14 (±0.02)
				30	0.99	0.15 (±0.04)	0.06 (±0.06)	0.13 (±0.02)
	EPO	0.99	0.10 (±0.01)	20	0.99	0.09 (±0.02)	0.02 (±0.01)	0.09 (±0.02)
				25	0.99	0.09 (±0.01)	0.03 (±0.02)	0.09 (±0.01)
				30	0.99	0.10 (±0.02)	0.04 (±0.03)	0.09 (±0.01)
25	SNV+2D	0.99	0.13 (±0.02)	20	0.98	0.37 (±0.05)	0.32 (±0.07)	0.18 (±0.04)
				25	0.98	0.15 (±0.03)	0.03 (±0.02)	0.14 (±0.03)
				30	0.99	0.14 (±0.01)	0.03 (±0.02)	0.14 (±0.02)
	EMSC	0.99	0.12 (±0.01)	20	0.99	0.21 (±0.07)	0.15 (±0.09)	0.13 (±0.01)
				25	0.98	0.15 (±0.04)	0.03 (±0.02)	0.15 (±0.04)
				30	0.99	0.14 (±0.02)	0.04 (±0.03)	0.13 (±0.02)
	EPO	0.99	0.09 (±0.00)	20	0.99	0.11 (±0.03)	0.07 (±0.03)	0.09 (±0.02)
				25	0.99	0.09 (±0.01)	0.03 (±0.01)	0.08 (±0.01)
				30	0.99	0.09 (±0.02)	0.03 (±0.02)	0.09 (±0.01)
30	SNV+2D	0.99	0.14 (±0.00)	20	0.97	0.80 (±0.13)	0.77 (±0.13)	0.21 (±0.04)
				25	0.98	0.28 (±0.04)	0.23 (±0.04)	0.15 (±0.03)
				30	0.99	0.14 (±0.01)	0.02 (±0.02)	0.14 (±0.01)
	EMSC	0.99	0.13 (±0.00)	20	0.99	0.18 (±0.10)	0.10 (±0.12)	0.13 (±0.03)
				25	0.99	0.14 (±0.01)	0.04 (±0.04)	0.12 (±0.01)
				30	0.99	0.13 (±0.01)	0.02 (±0.02)	0.12 (±0.01)
	EPO	0.99	0.09 (±0.00)	20	0.99	0.26 (±0.15)	0.23 (±0.16)	0.10 (±0.03)
				25	0.99	0.13 (±0.06)	0.08 (±0.07)	0.09 (±0.02)
				30	0.99	0.09 (±0.01)	0.02 (±0.01)	0.09 (±0.01)

**Table 3:** Performance comparison for SSC prediction of kiwifruit juice in the first overtone (870–1100 nm) region of the OH stretch of water at different temperatures.

With 10 mm Cuvette in the Second Overtone Region (870–1100 nm)								
N_cal_ = 72, N_val_ = 23								
Calibration				Validation				
T_cal_ [°C]	Method	r^2^_cv_	RMSECV	T_val_ [°C]	r^2^_p_	RMSEP	BIAS	SEP
All	SNV + 2D	0.98	0.17 (±0.01)	20	0.98	0.18 (±0.04)	0.06 (±0.05)	0.16 (±0.03)
				25	0.98	0.17 (±0.02)	0.05 (±0.02)	0.16 (±0.03)
				30	0.98	0.18 (±0.02)	0.04 (±0.02)	0.17 (±0.02)
20	SNV + 2D	0.98	0.20 (±0.01)	20	0.97	0.21 (±0.03)	0.07 (±0.03)	0.21 (±0.04)
				25	0.97	0.20 (±0.04)	0.06 (±0.03)	0.20 (±0.03)
				30	0.97	0.19 (±0.03)	0.05 (±0.03)	0.20 (±0.03)
	EMSC	0.99	0.14 (±0.01)	20	0.98	0.15 (±0.03)	0.04 (±0.02)	0.14 (±0.03)
				25	0.98	0.18 (±0.02)	0.07 (±0.04)	0.16 (±0.02)
				30	0.98	0.20 (±0.05)	0.10 (±0.07)	0.17 (±0.03)
	EPO	0.99	0.12 (±0.01)	20	0.99	0.13 (±0.01)	0.02 (±0.02)	0.13 (±0.01)
				25	0.98	0.14 (±0.02)	0.03 (±0.02)	0.14 (±0.02)
				30	0.98	0.17 (±0.03)	0.09 (±0.05)	0.15 (±0.02)
25	SNV+2D	0.98	0.20 (±0.01)	20	0.97	0.21 (±0.03)	0.07 (±0.03)	0.21 (±0.04)
				25	0.97	0.20 (±0.04)	0.06 (±0.03)	0.20 (±0.03)
				30	0.97	0.19 (±0.03)	0.05 (±0.03)	0.20 (±0.03)
	EMSC	0.99	0.15 (±0.01)	20	0.97	0.19 (±0.01)	0.07 (±0.03)	0.18 (±0.02)
				25	0.99	0.14 (±0.03)	0.04 (±0.04)	0.12 (±0.03)
				30	0.98	0.17 (±0.03)	0.06 (±0.06)	0.14 (±0.02)
	EPO	0.99	0.12 (±0.01)	20	0.97	0.21 (±0.02)	0.07 (±0.04)	0.19 (±0.03)
				25	0.99	0.13 (±0.02)	0.04 (±0.02)	0.12 (±0.01)
				30	0.98	0.24 (±0.05)	0.18 (±0.06)	0.15 (±0.03)
30	SNV+2D	0.98	0.19 (±0.01)	20	0.97	0.55 (±0.18)	0.51 (±0.18)	0.21 (±0.04)
				25	0.98	0.35 (±0.12)	0.29 (±0.13)	0.18 (±0.01)
				30	0.97	0.19 (±0.03)	0.06 (±0.03)	0.18 (±0.03)
	EMSC	0.98	0.17 (±0.02)	20	0.98	0.34 (±0.06)	0.29 (±0.06)	0.16 (±0.03)
				25	0.98	0.25 (±0.01)	0.18 (±0.03)	0.16 (±0.03)
				30	0.98	0.17 (±0.05)	0.05 (±0.03)	0.15 (±0.05)
	EPO	0.99	0.13 (±0.00)	20	0.98	0.14 (±0.02)	0.04 (±0.02)	0.14 (±0.02)
				25	0.99	0.14 (±0.01)	0.05 (±0.01)	0.13 (±0.01)
				30	0.99	0.12 (±0.01)	0.04 (±0.02)	0.12 (±0.01)

## Sample Availability

Samples of the compounds are not available from the authors.
